# Reporting of surrogate endpoints in randomised controlled trial reports (CONSORT-Surrogate): extension checklist with explanation and elaboration

**DOI:** 10.1136/bmj-2023-078524

**Published:** 2024-07-09

**Authors:** Anthony Muchai Manyara, Philippa Davies, Derek Stewart, Christopher J Weir, Amber E Young, Jane Blazeby, Nancy J Butcher, Sylwia Bujkiewicz, An-Wen Chan, Dalia Dawoud, Martin Offringa, Mario Ouwens, Asbjørn Hróbjartsson, Alain Amstutz, Luca Bertolaccini, Vito Domenico Bruno, Declan Devane, Christina D C M Faria, Peter B Gilbert, Ray Harris, Marissa Lassere, Lucio Marinelli, Sarah Markham, John H Powers, Yousef Rezaei, Laura Richert, Falk Schwendicke, Larisa G Tereshchenko, Achilles Thoma, Alparslan Turan, Andrew Worrall, Robin Christensen, Gary S Collins, Joseph S Ross, Rod S Taylor, Oriana Ciani

**Affiliations:** 1 MRC/CSO Social and Public Health Sciences Unit, School of Health and Wellbeing, University of Glasgow, Glasgow, UK; 2 Global Health and Ageing Research Unit, Bristol Medical School, University of Bristol, Bristol, UK; 3 Population Health Sciences, Bristol Medical School, University of Bristol, Bristol, UK; 4 Patient author, UK; 5 Edinburgh Clinical Trials Unit, Usher Institute, University of Edinburgh, Edinburgh, UK; 6 Bristol NIHR Biomedical Research Centre, Bristol, UK; 7 University Hospitals Bristol and Weston NHS Foundation Trust, Bristol, UK; 8 Child Health Evaluative Sciences, Hospital for Sick Children Research Institute, Toronto, ON, Canada; 9 Department of Psychiatry, University of Toronto, Toronto, ON, Canada; 10 Biostatistics Research Group, Department of Population Health Sciences, University of Leicester, Leicester, UK; 11 Women’s College Research Institute, Toronto, ON, Canada; 12 Department of Medicine, University of Toronto, Toronto, ON, Canada; 13 Science, Evidence, and Analytics Directorate, Science Policy and Research Programme, National Institute for Health and Care Excellence, London, UK; 14 Faculty of Pharmacy, Cairo University, Cairo, Egypt; 15 Department of Paediatrics, University of Toronto, Toronto, ON, Canada; 16 AstraZeneca, Mölndal, Sweden; 17 Centre for Evidence-Based Medicine Odense and Cochrane Denmark, Department of Clinical Research, University of Southern Denmark, Odense, Denmark; 18 Open Patient data Explorative Network, Odense University hospital, Odense, Denmark; 19 CLEAR Methods Centre, Division of Clinical Epidemiology, Department of Clinical Research, University Hospital Basel and University of Basel, Basel, Switzerland; 20 Oslo Centre for Biostatistics and Epidemiology, Oslo University Hospital, Oslo, Norway; 21 Bristol Medical School, University of Bristol, Bristol, UK; 22 Department of Thoracic Surgery, IEO, European Institute of Oncology IRCCS, Milan, Italy; 23 IRCCS Galeazzi-Sant’Ambrogio Hospital, Department of Minimally Invasive Cardiac Surgery, Milan, Italy; 24 University of Galway, Galway, Ireland; 25 Health Research Board-Trials Methodology Research Network, University of Galway, Galway, Ireland; 26 Department of Physical Therapy, Universidade Federal de Minas Gerais, Belo Horizonte, Brazil; 27 Fred Hutchinson Cancer Centre, Seattle, WA, USA; 28 St George Hospital and School of Population Health, University of New South Wales, Sydney, NSW, Australia; 29 Department of Neuroscience, Rehabilitation, Ophthalmology, Genetics, Maternal and Child Health, University of Genova, Genoa, Italy; 30 IRCCS Ospedale Policlinico San Martino, Genoa, Italy; 31 Department of Biostatistics and Health Informatics, Institute of Psychiatry, Psychology and Neuroscience, King’s College London, London, UK; 32 George Washington University School of Medicine, Washington, DC, USA; 33 Heart Valve Disease Research Centre, Rajaie Cardiovascular Medical and Research Centre, Iran University of Medical Sciences, Tehran, Iran; 34 Ardabil University of Medical Sciences, Ardabil, Iran; 35 Behyan Clinic, Pardis New Town, Tehran, Iran; 36 University of Bordeaux, Centre d’Investigation Clinique-Epidémiologie Clinique 1401, Research in Clinical Epidemiology and in Public Health and European Clinical Trials Platform & Development/French Clinical Research Infrastructure Network, Institut National de la Santé et de la Recherche Médicale/Institut Bergonié/Centre Hospitalier Universitaire Bordeaux, Bordeaux, France; 37 Charité Universitätsmedizin Berlin, Berlin, Germany; 38 Department of Quantitative Health Sciences, Lerner Research Institute, Cleveland Clinic, Cleveland, OH, USA; 39 McMaster University, Hamilton, ON, Canada; 40 Department of Outcomes Research, Anaesthesiology Institute, Cleveland Clinic, OH, USA; 41 Section for Biostatistics and Evidence-Based Research, the Parker Institute, Bispebjerg and Frederiksberg Hospital, Copenhagen and Research Unit of Rheumatology, Department of Clinical Research, University of Southern Denmark, Odense University Hospital, Odense, Denmark; 42 UK EQUATOR Centre, Centre for Statistics in Medicine, Nuffield Department of Orthopaedics, Rheumatology, and Musculoskeletal Sciences, University of Oxford, Oxford, UK; 43 Department of Health Policy and Management, Yale School of Public Health, New Haven, CT, USA; 44 Section of General Medicine, Department of Internal Medicine, Yale School of Medicine, New Haven, CT, USA; 45 Robertson Centre for Biostatistics, School of Health and Well Being, University of Glasgow, Glasgow, UK; 46 Centre for Research on Health and Social Care Management, Bocconi University, Milan 20136, Italy

## Abstract

Randomised controlled trials commonly use surrogate endpoints to substitute for a target outcome (outcome of direct interest and relevance to trial participants, clinicians, and other stakeholders—eg, all cause mortality) to improve their efficiency (through shorter trial duration, reduced sample size, and thus lower research costs), or for ethical or practical reasons. But reliance on surrogate endpoints can increase the uncertainty of an intervention’s treatment effect and potential failure to provide adequate information on intervention harms, which has led to calls for improved reporting of trials using surrogate endpoints. This report presents a consensus driven reporting guideline for trials using surrogate endpoints as the primary outcomes—the CONSORT (Consolidated Standards of Reporting Trials) extension checklist: CONSORT-Surrogate. The extension includes nine items modified from the CONSORT 2010 checklist and two new items. Examples and explanations for each item are provided. We recommend that all stakeholders (including trial investigators and sponsors, journal editors and peer reviewers, research ethics reviewers, and funders) use this extension in reporting trial reports using surrogate endpoints. Use of this checklist will improve transparency, interpretation, and usefulness of trial findings, and ultimately reduce research waste.

Evidence from well designed, conducted, and reported randomised controlled trials (referred to as trials in this article) assessing the effect of an intervention on the target outcome of interest (eg, all cause mortality) are required to determine the efficacy or effectiveness of interventions.^1^ Inadequate reporting of trials reduces their usefulness for decision making and, thus, contributes to the rising problem of research waste.^2^ ^3^ Using reporting guidelines has been shown to be successful in improving the usefulness of trial evidence and reduce research waste.^3^ The CONSORT (Consolidated Standards of Reporting Trials) statement is a 25 item checklist widely used for the reporting of parallel group trial reports.^4^ While the CONSORT checklist has improved the completeness of trial reports,^5^ it is not adequate for all types of trials. Consequently, CONSORT extensions (checklists with modified or new items) have been developed (eg, CONSORT-PRO (patient reported outcomes),^6^ CONSORT-Outcomes^7^). However, none of the existing extensions provides specific guidance for trials that use surrogate endpoints. Surrogate endpoints act as a substitute in trials for target outcomes.^8^ ^9^ Table 1 lists some examples of surrogate endpoints applicable in trials.

**Table 1:** Examples of surrogate endpoints in randomised controlled trials

Item	Example 1^10^	Example 2^11^	Example 3^12^
Domain-surrogate endpoint	Blood pressure	Tumour response	Body mass index
Measurement variable or specific measurement	Daytime ambulatory systolic blood pressure	Assessed by independent central review according to Response Evaluation Criteria in Solid Tumors, version 1.1, with use of contrast enhanced computed tomography or magnetic resonance imaging	Calculated from weight and height obtained from electronic health record
Specific metric	Change from baseline	Value at time point	Change from baseline
Method of aggregation	Continuous outcome: mean	Binary outcome: frequency (%)	Per cent from the median body mass index for age and sex
Time point	2 months after randomisation	Median 15.3 months	2 years after parent’s consent or enrolment into the study
Target outcome(s) as stated by authors	Stroke, coronary heart disease, heart failure, all cause mortality	Overall survival	Diabetes, liver disease, asthma, heart disease, cancer, lower health related quality of life, behaviour problems, psychosocial dysfunction

First five rows represent core elements of a defined outcome adapted from Butcher et al,^7^ Chan et al,^13^ Mayo-Wilson et al,^14^ and Zarin et al.^15^

Surrogate endpoints are frequently used to improve trial efficiency (eg, to shorten duration of follow-up, reduce sample size, and, thus, lower overall trial costs) among other feasibility, practical, ethical, and scientific reasons.^16^ Dependent on the disease or health area and definitions of a surrogate endpoint, it has been estimated that 20-78% of trials use surrogate endpoints as primary outcomes.^17^ ^18^ ^19^ ^20^ However, in the absence of data on target outcomes, the use of surrogate endpoints in trials can be controversial and have fundamental limitations for clinical and policy decision making such as an increase of the uncertainty of the intervention’s true effect on target outcome (and clinical efficacy or effectiveness, and cost effectiveness) and failure to provide adequate information on intervention harms, given their typically smaller sample size and shorter follow-up period.^16^ Consequently, there have been calls for better reporting of trials that rely on surrogate endpoints, including an explicit statement and rationale for the use of a surrogate endpoint and consideration of their potential limitations.^20^ ^21^ ^22^ ^23^ Considering the ongoing inadequacies in reporting trials using surrogate endpoints, the SPIRIT/CONSORT-Surrogate project was formed to develop extensions for SPIRIT and CONSORT for trials using a surrogate endpoint as a primary outcome (video 1). The SPIRIT-Surrogate extension is presented in Manyara et al.^24^ In this article, we present the CONSORT-Surrogate extension checklist along with an elaboration and explanation document. Table 2 provides a glossary of terminology used in the extension.

**Table 2:** Glossary of terminology used in the CONSORT-Surrogate extension

Term	Definitions
Composite outcome	Outcome consisting of two or more component outcomes (eg, proportion of participants who died or had a non-fatal stroke). Participants who have experienced any one of the events specified by the components are considered to have experienced the composite outcome.^25^
CONSORT	Consolidated Standards of Reporting Trials. Reporting checklist for completed randomised controlled trials.
CONSORT-Surrogate	Modified CONSORT checklist used to report trials using surrogate endpoints as primary outcomes.
Primary outcome	Predefined outcome that trial teams consider to be the most important and feasible in evaluating the effectiveness of an intervention, which informs sample size calculation and trial conclusions; sometimes referred to as primary endpoint^7^ ^26^
Secondary outcome	Outcome prespecified in a trial protocol to measure additional intervention effects^6^; can be confirmatory or exploratory based on the design of the study (ie, statistical adjustment for increased false positive results)
SPIRIT	Standard Protocol Items: Recommendations for Interventional Trials. Reporting checklist for completed randomised controlled trial protocols
SPIRIT-Surrogate	Modified SPIRIT checklist used to report trial protocols using surrogate endpoints as primary outcomes
Surrogate endpoint*†	Endpoint that is used in trials as a substitute for a direct measure of how a patient feels, functions, or survives; it does not measure the actual clinical benefit of primary interest, but is expected to predict the treatment effects on clinical benefit or harm based on epidemiological, therapeutic, pathophysiological, or other scientific evidence^8^
Target outcome	Outcome of direct interest and relevance to trial participants, patients, clinicians, trialists, or other stakeholders^27^

* SPIRIT/CONSORT-Surrogate extension project research, including an e-Delphi and e-survey, investigated the definition of a surrogate endpoint among different stakeholders: trial participants, clinicians, trialists, regulators, and payers. The results of this research and these definitional considerations are reported in detail elsewhere.^27^

† Other descriptive terms used with “surrogate” are “outcome,” “marker,” “measure,” “observation,” or “parameter.” Can also be referred to as “early,” “replacement,” “proxy,” “substitute endpoints,” “outcomes,” “measures,” and “markers” in the literature.

**Summary points**

Randomised controlled trials often rely on surrogate endpoints to replace a target outcome of interest, particularly in the regulatory approval and health technology assessment of drugs and biological agents
Use of surrogate endpoints in trials might be misleading in terms of claims of intervention efficacy or effectiveness on target outcomes, and by providing limited information on harms
This article describes the CONSORT-Surrogate extension, a guideline to improve reporting of trial reports using a surrogate endpoint as a primary outcome to consequently inform better patient care, healthcare decisions, and policies
Trial authors, journal editors, and reviewers should use the CONSORT-Surrogate extension to improve reporting relevant protocols to enhance completeness, transparency, replicability of methods, interpretation, and usefulness of findings

## Scope and use of CONSORT-Surrogate

Box 1 summarises the scope and use of the CONSORT-Surrogate extension. The extension should be used to report all trial types and phases that use surrogate endpoints (based on any definition) as primary outcome(s) including when a surrogate endpoint is used as part of a composite outcome. Given that primary outcomes drive evaluation of interventions and trial conclusions, the focus of the extension is on this aspect. The extension provides the minimum recommended items to report, but authors can provide additional information that helps with transparency of surrogate endpoint trials and interpretation of results. Importantly, the extension does not mandate trial teams to change their design or plans to fit with recommended items: authors should just be explicit about what was done or planned but are strongly encouraged to consider implementing all items, when possible. Box 1 presents more aspects on the scope and use of the extension. Appendix table A1 presents key methodological considerations of the design and the reporting of surrogate endpoints in trial reports, that inform the extension items.

Box 1 **Summary of scope and use of CONSORT-Surrogate extension**

**Eligibility for use**

All intervention randomised controlled trials using surrogate endpoints (based on any definition) as primary outcome(s). Includes instances when surrogate endpoints are part of a primary composite outcome.

**Minimum requirement**

The extension is the minimum set of items to be reported but authors can provide more information for improved transparency, clarity, and interpretation of findings.

**Surrogate validation methods are out of scope**

The appraisal of surrogate validation methods or metrics to use or cite is out of the scope of this extension.

**Target outcome(s)**

Trial teams should consider collecting target outcomes (as secondary outcome(s)) and reporting their intervention effects. Such information can support subsequent surrogate endpoint validation analyses and assessment of potential intervention harms.

**Flexibility in order of reporting items**

Items can be combined or reported in different sections to those items suggested in the extension. The specific item sections are recommendations rather than requirements.

**Extrapolation of extension items**

The extension was developed for trials, but could be relevant to report non-randomised trials, observational studies, and other studies using surrogate endpoints.

CONSORT=Consolidated Standards of Reporting Trials.

## Development of CONSORT-Surrogate extension

Development of the CONSORT-Surrogate extension, undertaken alongside the SPIRIT-Surrogate extension, followed four phases informed by the EQUATOR (Enhancing the QUAlity and Transparency Of health Research) network guidance for developing health reporting guidelines.^28^ The development was pre-registered on the EQUATOR network website^29^ and the protocol published.^30^ Phase 1 involved literature reviews aimed at synthesising reporting items of trials using surrogate endpoints from current literature and identifying surrogate content experts (scoping review); and identifying trial investigators of recent trials using surrogate endpoints as primary outcomes for invitation to an e-Delphi survey (targeted review). The protocol for the literature reviews has been published elsewhere.^31^ The scoping review search was undertaken between March and May 2022 and 90 documents included after screening. Data on definitions, limitations, acceptability, and guidance were extracted and used to generate 17 trial reporting items, the findings of the scoping review including the 17 generated items have been published elsewhere.^16^ After a project team discussion, 13 items were taken forward for rating in the e-Delphi survey.

Phase 2 involved rating of potential reporting items in a two round, e-Delphi survey using a 9 point Likert scale (1-3: not important; 4-6: important but not critical; 7-9: critical) on the DelphiManager software (version 5.0), maintained by the COMET initiative (Core Outcome Measures in Effectiveness Trials; https://www.comet-initiative.org/delphimanager/). The first round was open from 24 August to 10 October 2022; and the second round from 31 October to 11 December 2022. Participants were identified through various ways: contacting authors of relevant articles from the literature reviews; project team professional contacts; calls for participants made in conferences and meetings, social media, and distributed through professional organisations and networks (listed in appendix 2).

A total of 212 eligible participants registered to participate, with 195 (92%) rating the items in the first round and 176 (83%) in the second round. Participants represented 30 countries and encompassed a multidisciplinary group of stakeholders, including trial investigators, trial methodologists (including statisticians), trial managers, clinicians and allied health professionals, surrogate content experts, journal editors, patient and public partners, regulators, and payers or health technology assessment experts, ethics committee and funding panel members. Appendix tables A2, A3, A4, and A5 list the characteristics of participants.

Consensus thresholds for inclusion of items were ≥70% score of 7-9 and <15% score of 1-3, consensus thresholds for exclusion were ≥70% score of 1-3 and <15% score of 7-9, and no consensus for inclusion or exclusion was the failure to achieve either threshold.^30^ Thirteen items for CONSORT-Surrogate were rated in round one and 14 items in round two (additional item was suggested by participants in round one). Eight items achieved consensus thresholds in round one and a further two items in round two while there was no consensus for four items after both rounds (appendix table A6).

Phase 3 was a hybrid consensus meeting held on 13 and 14 March 2023 at the University of Glasgow, Glasgow, UK and via Zoom. Meeting delegates included 13 project team members and an invited subset of 20 stakeholders who had participated in the e-Delphi survey. The four items that did not reach consensus in the e-Delphi survey were discussed and voted on (using https://www.mentimeter.com/). Consensus was predefined as ≥70% voting to include or exclude an item. All four items achieved consensus: two for inclusion and two for exclusion (appendix table A7). For items that reached consensus, meeting delegates also fine-tuned wording and merging of items and discussed free text comments provided from e-Delphi surveys. 

Phase 4 is an ongoing knowledge translation that includes dissemination and implementation of extensions. Dissemination efforts have included publication of short articles to publicise the project^32^ ^33^ ^34^ ^35^ ^36^; publication of protocols^30^ ^31^; and presentations in meetings and conferences. The completed checklist was piloted by eight trial investigators who had conducted at least one trial by providing them with published trial and asking them to note whether extension items were reported. All items were clear, and no changes were made as a result of the pilot exercise.

## Structure of the CONSORT-Surrogate extension

The extension consists of a checklist that is accompanied by an explanation and an elaboration section to provide rationale and clarification on modified or new items. Additionally, exemplars reporting the extension items are provided. For items that have not been extended, users should refer to the CONSORT 2010 explanation and elaboration document.^4^ We used 12 published trial reports to provide at least one example of reporting in each of the 11 CONSORT-Surrogate extension items. Eight (67%) of the trial reports used as examples were identified from a targeted review of trial protocols published between January 2017 and June 2022 in six general medical journals and the rest were identified from exploratory searches in PubMed database. The example text in this article includes a “ref” in superscript to indicate cited references within the examples. We have supplemented some examples by adding terms and recommendations to enhance their use. Abbreviations have also been spelt out in the examples where necessary. Use of any of the examples does not imply our support for the trial findings, conclusions, or endorsement of the interventions evaluated. Furthermore, it is not possible to identify and list examples from all disease and research areas that should use this extension. Therefore, trial teams can use examples provided as a guide on how the items can be reported in their own disease or research area. The identification of examples for nearly all extension items demonstrates the feasibility of implementing the extensions in trial reports.

Despite extensive efforts, which included reviewing trial reports from targeted reviews and seeking exemplars from colleagues, we were unable to find an example that effectively implemented one item: informing participants that the trial used a surrogate endpoint. Therefore, together with patient and public partners who are coauthors of this extension (DS, RH, SM, AW), we have modified a quote from a published protocol to demonstrate how this item can be reported in a trial report (item 26a.1).

## CONSORT-Surrogate extension

Table 3 compares the CONSORT 2010 checklist with the extension items in the CONSORT-Surrogate checklist. Appendix 3 presents a combined CONSORT 2010 and CONSORT-Surrogate checklist, which can be downloaded and completed separately.

**Table 3:** Comparison of items from CONSORT 2010 and CONSORT-Surrogate extension

Section/topic	Item No	CONSORT checklist item	CONSORT-Surrogate extension item
**Title and abstract**			
	1a	Identification as a randomised trial in the title	—
	1b	Structured summary of trial design, methods, results, and conclusions (for specific guidance, see CONSORT for abstracts)	1b.1 State (a) that the primary outcome is a surrogate endpoint, and (b) the target outcome(s) whose intervention effect is being substituted for.
**Introduction**			
Background and objectives	2a	Scientific background and explanation of rationale	2.1 State (a) that the primary outcome is a surrogate endpoint, and (b) the target outcome(s) whose intervention effect is being substituted for.
	2b	Specific objectives or hypotheses	
**Methods**			
Trial design	3a	Description of trial design (such as parallel, factorial) including allocation ratio	—
	3b	Important changes to methods after trial commencement (such as eligibility criteria), with reasons	—
Participants	4a	Eligibility criteria for participants	—
	4b	Settings and locations where the data were collected	—
Interventions	5	The interventions for each group with sufficient details to allow replication, including how and when they were actually administered	—
Outcomes	6a	Completely defined prespecified primary and secondary outcome measures, including how and when they were assessed	6a.1 State the practical or scientific reason(s) for using a surrogate endpoint as a primary outcome
			6a.2 Justification for selected surrogate: (a) evidence (or lack of evidence) of surrogate endpoint validation; and (b) evidence (or lack of evidence) of validity being specific to setting and context used (eg, intervention; disease; population).
	6b	Any changes to trial outcomes after the trial commenced, with reasons	—
Sample size	7a	How sample size was determined	7a.1 Clarify if sample size was estimated to demonstrate that a minimum effect on the surrogate endpoint would be predictive of a benefit on the target outcome(s).
	7b	When applicable, explanation of any interim analyses and stopping guidelines	—
Randomisation:			
Sequence generation	8a	Method used to generate the random allocation sequence	—
	8b	Type of randomisation; details of any restriction (such as blocking and block size)	—
Allocation concealment mechanism	9	Mechanism used to implement the random allocation sequence (such as sequentially numbered containers), describing any steps taken to conceal the sequence until interventions were assigned	—
Implementation	10	Who generated the random allocation sequence, who enrolled participants, and who assigned participants to interventions	—
Blinding	11a	If done, who was blinded after assignment to interventions (eg, participants, care providers, those assessing outcomes) and how	—
	11b	If relevant, description of the similarity of interventions	—
Statistical methods	12a	Statistical methods used to compare groups for primary and secondary outcomes	—
	12b	Methods for additional analyses, such as subgroup analyses and adjusted analyses	—
**Results**			
Participant flow (a diagram is strongly recommended)	13a	For each group, the numbers of participants who were randomly assigned, received intended treatment, and analysed for the primary outcome	—
	13b	For each group, losses, and exclusions after randomisation, together with reasons	—
Recruitment	14a	Dates defining the periods of recruitment and follow-up	—
	14b	Why the trial ended or was stopped	—
Baseline data	15	A table showing baseline demographic and clinical characteristics for each group	—
Numbers analysed	16	For each group, number of participants (denominator) included in each analysis and whether the analysis was by original assigned groups	—
Outcomes and estimation	17a	For each primary and secondary outcome, results for each group, and the estimated effect size and its precision (such as 95% confidence interval)	17a.1 If the primary outcome is a composite outcome that includes a surrogate endpoint; report the intervention effect on all components.
	17b	For binary outcomes, presentation of both absolute and relative effect sizes is recommended	—
Ancillary analyses	18	Results of any other analyses performed, including subgroup analyses and adjusted analyses, distinguishing prespecified from exploratory	—
Harms	19	All important harms or unintended effects in each group (for specific guidance, see CONSORT for harms)	—
**Discussion**			
Limitations	20	Trial limitations, addressing sources of potential bias, imprecision, and, if relevant, multiplicity of analyses	—
Generalisability	21	Generalisability (external validity, applicability) of the trial findings	—
Interpretation	22	Interpretation is consistent with results, balancing benefits and harms, and considering other relevant evidence	22.1 Interpretation of findings of the trial in the context of using a surrogate primary endpoint, including its known validity for intervention effects on the target outcome and the potential benefit-risk assessments of the tested intervention for participants.
			22.2 Comment on whether the trial design (including sample size and follow-up period), given the use of a surrogate endpoint, adequately captures the potential harms of the intervention being tested.
			22.3 State what the plans are to conduct subsequent analyses/studies to verify current findings on the target outcome(s).
**Other information**			
Registration	23	Registration number and name of trial registry	—
Protocol	24	Where the full trial protocol can be accessed, if available	—
Funding	25	Sources of funding and other support (such as supply of drugs), role of funders	—
	26	New items	26.1 State whether and how trial participants were engaged and informed before enrolment that the trial was designed to evaluate an intervention’s effect using a surrogate endpoint. 26.2 If surrogate endpoint and target outcome data were collected in the trial, state the open access arrangements for the data for future secondary research.

Appendix 3 presents a combined CONSORT 2010 and CONSORT-Surrogate checklist, which can be downloaded and completed separately.

CONSORT=Consolidated Standards of Reporting Trials.

## Title and abstract

### Items 1b (extended)

#### CONSORT 2010 item 1b

Structured summary of trial design, methods, results, and conclusions.^4^

For specific guidance, see CONSORT for abstracts.^37^

#### CONSORT-Surrogate extension item 1b.1

State (a) that the primary outcome is a surrogate endpoint, and (b) the target outcome(s) whose intervention effect is being substituted for.

### Examples of CONSORT-Surrogate item 1b.1

#### Example 1 

“The primary outcome was the peak change of urinary neutrophil gelatinase-associated lipocalin within 48 h, a surrogate marker [endpoint] of kidney injury.”^38^ (We have added the word “endpoint” and recommend its use.)

#### Example 2

“To evaluate the effects of the dipeptidyl peptidase-4 (DPP-4) inhibitor linagliptin on aortic pulse wave velocity (PWV) as a surrogate [endpoint] marker of arterial stiffness and early atherosclerosis in people with early type 2 diabetes.”^39^ (We recommend the use of the term “surrogate endpoint” rather than “marker”.)

#### Explanation

Well written trial abstracts provide an initial assessment to readers to decide whether to read or access the full report and, in some cases, they can solely inform healthcare decisions.^37^ ^40^ Explicit mention of certain items in abstracts is important for database indexing^37^ and consequent retrieval of trials for secondary research such as surrogate endpoint validation. Despite their importance, space restrictions require that abstracts only present the key information of a trial.^41^ In addition to using CONSORT for abstracts,^37^ trial authors should be explicit about the use of a surrogate endpoint as a primary outcome and the target outcome being substituted for. Given the varying structures of abstracts and limited space,^40^ ^41^ authors can report this item in various ways, as seen from examples provided.

## Introduction

### Background and objectives (extended)

#### CONSORT 2010 item 2a 

Scientific background and explanation of rationale.

See CONSORT 2010.^4^

#### CONSORT 2010 item 2b

Specific objectives or hypotheses. 

See CONSORT 2010.^4^

#### CONSORT-Surrogate extension item 2.1

State (a) that the primary outcome is a surrogate endpoint, and (b) the target outcome(s) whose intervention effect is being substituted for.

### Example of CONSORT-Surrogate extension item 2.1

“PWV [aortic pulse wave velocity] is an integrated index of arterial function and structure and hence a [surrogate endpoint] marker of early atherosclerosis ^ref^. A higher PWV is associated with a more stiffened artery and an increased risk of CV [cardiovascular] events^ref^.”^39^ (We have added the word “surrogate” in the example and recommend its use when reporting the item. We also recommend use of the term “surrogate endpoint” and specific citation of the reference supporting the validity of surrogate endpoint (see explanation of item 6a.2).)

#### Explanation

The introduction outlines the reasons for conducting the trial by summarising current evidence and knowledge gaps being filled^4^ ^40^; see the REPORT guide for more information on introduction content and structure.^40^ The introduction gives readers a general outline of the trial report^4^ ^40^ and allows journal editors and reviewers to assess the importance of a trial report.^40^ Therefore, authors need to be explicit about using a surrogate endpoint and the target outcome for whose treatment effect is substituted for. Given that introduction sections in final trial publications can be shorter than for protocol publications,^41^ a brief statement of the primary outcome being a surrogate endpoint and the associated target outcome would be sufficient for this item. Authors can outline more details on the surrogate endpoint(s) selected, including their justification in the methods section (see items 6a.1 and 6a.2). However, authors could summarise these items in the introduction if it gives readers a better context or importance of the trial. Finally, because introductions have different structures and word lengths, authors can report the item when reporting either CONSORT 2010 item 2a or item 2b.

## Methods

### Outcomes

#### CONSORT 2010 item 6a (extended)

Completely defined prespecified primary and secondary outcome measures, including how and when they were assessed. 

See CONSORT 2010^4^ and CONSORT-Outcomes extension.^7^

#### CONSORT-Surrogate extension item 6a.1

State the practical or scientific reason(s) for using a surrogate endpoint as a primary outcome.

#### CONSORT-Surrogate extension item 6a.2

Justification for selected surrogate: (a) evidence (or lack of evidence) of surrogate endpoint validation; and (b) evidence (or lack of evidence) of validity being specific to setting and context used (eg, intervention; disease; population).

### Example of CONSORT-Surrogate item 6a.1

“We used surrogate endpoints for this trial because of a number of practical constraints, including the trial cost, rapidly evolving evidence in this field, and concern about the feasibility of conducting a long-term intervention in a vulnerable population. However, the endpoints selected have been validated as having prognostic significance for CVD [cardiovascular] events.”^42^

#### Explanation

Given limitations associated with surrogate endpoints,^16^ authors should inform readers of the scientific or practical reason(s) for using them. A commonly cited reason for use of surrogate endpoints is trial efficiency: shorter follow-up and smaller sample size. This use can be ideal for early phase trials where the focus is aimed at demonstrating biological activity and informing the need for future trials powered on target outcomes.^16^ Also, primary prevention trials can require a long time to accrue, and trials of rare diseases often have access to only small trial populations.^16^ Additionally, surrogate endpoints have been widely used in regulatory approval settings as part of expedited or accelerated approval for conditions with high unmet medical need in serious and life threatening diseases.^8^ ^16^ ^43^ Further, target outcomes might not be ideal in certain interventional contexts, for example, participant reported outcomes in paediatric trials can be challenging^44^ in newborn babies or very young children (aged <7 years) where observer reported outcomes are needed. The practical or scientific reasons for using surrogate endpoints highlighted here and elsewhere^16^ might not be exhaustive.

Reporting this item provides readers with a justification of using surrogate endpoint(s) as a primary outcome and contextualising the importance of the trial. However, adequate reporting of this item does not preclude authors from addressing item 6a.2 on the validity of the surrogate endpoint selected (see explanation for item 6a.2).

### Examples of CONSORT-Surrogate item 6a.2

#### Example 1

“The primary end point for these trials was chosen in agreement with the US Food and Drug Administration. Although no published data specifically document overt clinical benefits related to a 30% or greater reduction of PTH [parathyroid hormone], several observational studies have shown that PTH concentrations greater than 600 pg/mL [as a surrogate endpoint] are associated with higher rates of [target outcomes] death, cardiovascular events, and fracture than PTH concentrations in the range of 150 to 300 pg/mL.^refs^”^45^ (We have added words to the quote in square brackets and recommend their use when reporting the item.)

#### Example 2

“The primary efficacy endpoint was the change in daytime ambulatory systolic blood pressure from baseline to 2 months. Systolic blood pressure is a validated surrogate endpoint for prediction of cardiovascular events and mortality based on a meta-analysis of 123 blood pressure lowering drug trials, with 613,815 participants demonstrating a strong association between the treatment effect of systolic blood pressure and cardiovascular events ^ref^. Specifically meta-regression showed relative risk reductions for major cardiovascular disease events (P<0.0001), stroke (P<0.0001), heart failure (P<0.0001), and all-cause mortality (P=0.014) to be proportional to the magnitude of the systolic blood pressure reduction achieved. However, risk reductions for various diseases differed across drug classes more evidence is needed to establish that validity of blood pressure lowering to predict for benefit in cardiovascular events and mortality holds when renal denervation is used.” (This example was written by the authors from a published trial^10^ and using the meta-analysis^46^ cited by the trial that reported a strong association in the treatment effect on the surrogate endpoint (difference in systolic blood pressure) and the target outcome (relative risk for cardiovascular and all case mortality) across randomised controlled trials of interventions using blood pressure lowering drugs, see explanation for item 6a.2.)

#### Explanation

Surrogate endpoints should be validated before they are used. Validation is determining whether the intervention’s effect on the surrogate endpoint predicts the intervention effect on the target outcome.^47^ ^48^ While a detailed discussion of surrogate validation is beyond the scope of this extension, we signpost readers to several articles on surrogate validation methods,^47^ ^48^ ^49^ ^50^ ^51^ ^52^ ^53^ ^54^ ^55^ ^56^ frameworks for evaluating validity of evidence,^21^ ^57^ ^58^ ^59^ and recently, a checklist to report surrogate validation.^60^ In brief, validation should demonstrate both a strong association of the surrogate endpoint and target outcome (the so-called individual level association), and should demonstrate that the treatment effect on the surrogate must be tightly correlated with the treatment effect on the target outcome (the so-called trial level association).^47^ ^48^

For instance, example 1 for item 6a.2 fails to achieve this desired level of evidence, based on an observational association between PTH (parathyroid hormone; surrogate endpoint) and the target outcomes of mortality and major clinical events. In contrast, example 2 cites the association in treatment effect between the surrogate of systolic blood pressure and target outcome of mortality, based on a meta-analysis (regression) of randomised controlled trials. To fully judge the strength of validation for the validity of a surrogate endpoint, authors should provide some key meta-regression metrics, that is: the slope coefficient (and 95% confidence interval) of the linear relation between the treatment effect of the surrogate and the target outcome, the strength of the association such as Spearman’s correlation coefficient (ρ) or R^2^, and the surrogate treatment effect or prediction intervals (see item 7a.1). Illustration of these metrics for blood pressure and cardiovascular events can be found in the article by Lassere et al.^58^

Surrogate endpoint validation in trials needs to be better reported. An analysis of 626 trials published in 2005 and 2006 found that only 34% (37/109) of trial reports that used a surrogate endpoint as a primary outcome discussed its validity.^61^ In cancer, where several surrogate validation studies have been published, a systematic review indicated relatively poor validity of surrogate endpoints: 52% of surrogate endpoints used in trials had a low correlation in their treatment effect with the target outcome of overall survival (r ≤0.7), with only 23% demonstrating a high correlation (r ≥0.85). Surrogate validation models often provide the opportunity to predict the treatment effect on the target outcome in new trials for which the effect on the surrogate endpoint has been estimated. It is therefore important to quantify the accuracy of the predictions made.^60^ Leave-one-out cross validation and even external validation with new trials published after the model was fitted or trials whose individual patient data were not available for model estimation, are essential to assess the model’s predictive performance and calibration.^62^ Trial authors should therefore be explicit on the surrogate endpoint validity evidence (or lack of it). Over the years, many statistical approaches to surrogate validation have been proposed^54^ ^63^ (some of which are summarised in box 2). The approach underpinning the selection of the surrogate endpoint should be presented in detail.

Box 2 **Summary of statistical approaches for surrogate endpoint validation**

Selected and non-exhaustive statistical methods and general approaches for evaluating the validity of surrogate endpoints in the assessment of treatment efficacy that have emerged over the past four decades.

**Prentice’s criteria^53^**

In pioneering work published in 1989, Prentice proposed three criteria for valid hypothesis testing extrapolation (rejecting the null hypothesis of no treatment effect on the surrogate endpoint implies rejecting the null hypothesis of no treatment effect on the target outcome):

- The effect of the surrogate endpoint on the true endpoint does not vary with randomisation group;
- The surrogate endpoint affects the true endpoint;
- The effect of treatment on the surrogate endpoint changes the average effect of treatment on true endpoint.

The Prentice criteria remains conceptually important but of limited usefulness in practice.

**Principal stratification^64^**

This method maintains that causal effects should be the basis for surrogate endpoint evaluations, where the causal effect is a comparison between treatment groups of the potential outcomes on the same set of individuals. Two requirements are needed for surrogate validity: causal necessity, which requires that an effect of treatment on the target outcome can only exist if treatment has also affected the surrogate; and statistical generalisability, which requires good predictive performance of the surrogate for the target outcome in a future study in which only the surrogate is observed.

**Meta-analytical regression based approach^47^ ^65^**

This approach relies on two stage, joint modelling of the surrogate and target outcome in a multi-trial (randomised trials) setting. Surrogacy is established on the basis of the coefficient of determination between the surrogate and target outcome at the individual patient level (individual level R^2^), and the coefficient of determination between the treatment effect on the surrogate and on the target outcome at the trial level (trial level R^2^). Alternatively, the surrogate threshold effect has been proposed as a practical measure to define the minimum level of treatment effect required on the surrogate to conclude that a significant treatment effect would also be present on the target outcome.^66^ Extensions of these meta-analytical methods based on information theory have been proposed as the preferred approach under the causal association paradigm.^67^

**Bayesian approaches**

While a bayesian approach will be readily applicable to all the methodologies outlined above, the most commonly used models are the meta-analytical fixed (independent) effects model proposed by Daniels and Hughes^63^ and a bayesian random effects meta-analysis to model trial level effects on the target outcome and surrogate endpoint.^51^ More recently, bayesian multivariate meta-analytical methods to take into account the association between the treatment effects on the surrogate and target outcomes have been proposed specifically for regulatory and reimbursement decision making.^51^

Additionally, evidence of surrogate validity in one trial context (eg, sufficiently similar population, intervention, disease, control, and setting) might not generalise to another.^16^ For example, a systematic review of studies evaluating the validity of progression-free survival as a surrogate endpoint for overall survival found that trial level validity varied across the intervention evaluated, cancer localisation, and stage.^68^ The magnitude of weight loss assessed using body mass index, which predicts a morbidity or mortality benefit, often depends on disease or obesity related complications, the individual’s age, and their baseline obesity level.^69^ ^70^ Therefore, trial investigators should justify the surrogate endpoint based on evidence of surrogate validity (or lack of it) in the context used (see example 2 for item 6a.2 on validity, for being specific to different diseases but with acknowledgement of lack of evidence to the specific intervention being tested).

## Sample size

### *CONSORT 2010 item 7a (extended)*

How sample size was determined.

See CONSORT 2010.^4^

### CONSORT-Surrogate extension item 7a.1

Clarify if sample size was estimated to demonstrate that a minimum effect on the surrogate endpoint would be predictive of a benefit on the target outcome(s).

#### *Examples of CONSORT-Surrogate item 7a.1*

#### Example 1

“Because previously published data suggested a low overall incidence of CA-AKI [contrast associated-acute kidney injury] ^ref^ at our centre, we chose NGAL [neutrophil gelatinase-associated lipocalin] as primary outcome parameter. A formal power calculation was not performed for the primary endpoint of this exploratory study, because of a lack of suitable data on preventive therapy studies with rhC1INH at time of study design and therefore the use of potentially poor estimates of parameters for sample size calculations. In analogy to previous interventional studies using different prophylactic regimens ^refs^ and similar surrogate parameters of renal function, we calculated that 40 subjects are required in each study arm to allow for the detection of a difference in mean urinary peak NGAL concentration of 100 ng/mL assuming a standard deviation of 150 ng/mL, a power of 80%, and a 2-sided type 1 error of 5%. This difference has been shown to be predictive of [the target outcome:] AKI ^ref^.”^38^ (We have added words in square brackets and recommend their use. Given the exploratory context of this trial, authors use metrics from an observational study; however, trial teams should aim to use of metrics drawn directly from surrogate validation studies.)

#### Example 2

“The assumptions for the power calculation (threshold of a 40-m increase as the [surrogate threshold effect] minimal clinically important improvement in 6-minute walk test distance, with an SD [standard deviation] of 80m) were based on (1) a meta-regression of prior randomized clinical trials in patients with pulmonary arterial hypertension ^ref^ (due to the lack of such data in patients with HFpEF [heart failure with preserved ejection fraction]) and (2) clinical consensus among members of the trial’s steering committee.”^71^ (We recommend using the term “surrogate threshold effect” rather than “minimal clinically important improvement,” which is consistent with the cited surrogate validation study.)

#### Explanation

Trial sample size determination must be appropriately justified and adequately reported including details of the target effect size and allowance for sample trial attrition in the outcome.^4^ ^72^ Trials with a primary outcome that is a surrogate endpoint should consider their choice of a target effect size based on metrics of surrogate validity. For example, a commonly reported validation metric is the minimum treatment effect on the surrogate endpoint necessary to predict a treatment benefit on the target outcome known as a surrogate threshold effect.^58^ ^66^ The concept of surrogate threshold effect was used in example 2 of item 7a.1, although the authors acknowledge that this is derived from a different patient population (owing to data unavailability for their trial population).^71^ In contrast, in example 1 of item 7a.1,^38^ other metrics of surrogate validity are used—justification of the difference in the surrogate endpoint that is predictive of the target outcome is from a prospective study that used cut-off thresholds derived from a receiver operating curve.^73^

In some instances, owing to the absence of previous surrogate endpoint validation, it might not be possible for authors to use surrogate validity metrics formally to determine the sample size. However, trial investigators could consider prospectively validating their chosen surrogate endpoint if the data are available (see item 26.2). Furthermore, given that surrogate endpoints are mainly used improve trial efficiency (ie, allow for smaller sample size compared to using target outcomes), authors are encouraged to determine the sample size for both the surrogate endpoint and target outcome. If the sample size based on treatment effect on the target outcome is the same as (or smaller than) what the surrogate endpoint would be, then sufficient justification for the choice of surrogate as the primary outcome should be provided. Finally, whether validity metrics are used or not, authors should discuss the interpretation of findings in the context of using a surrogate endpoint and its known validity (see item 22.1), including how the predicted effect on the target outcome and its uncertainty (reflected by its confidence interval) has been derived.

## Results

### Outcomes and estimation

#### CONSORT 2010 item 17a (extended)

For each primary and secondary outcome, results for each group, and the estimated effect size and its precision (such as 95% confidence interval). 

See CONSORT 2010.^4^

#### CONSORT-Surrogate extension item 17a.1

If the primary outcome is a composite outcome that includes a surrogate endpoint; report the intervention effect on all components.

### Examples of CONSORT-Surrogate item 17a.1

See table 4 and table 5 for examples.

**Table 4:** Reporting individual components of an example composite measure: the American College of Rheumatology (ACR) response. Table generated using results from van de Putte et al,^74^ with permission from BMJ Publishing Group

Measure	Adalimumab 40 mg every 2 weeks (n=113)	Placebo (n=110)
**Composite ACR response (%)**		
ACR 20	46.0	19.1
ACR 50	22.1	8.2
ACR 70	12.4	1.8
**Individual measures—absolute change (% change)**		
Tender joint count (0-68)	−13.6 (−37.4)	−6.6 (−9.5)
Swollen joint count (0-66)	−8.5 (−37.0)	−2.4 (−7.4)
Patient assessment of pain (VAS 0-100)	−27.6 (−37.7)	−11.0 (−11.4)
Patient global assessment of disease activity (VAS 0-100)	−27.9 (−38.9)	−10.6 (−7.9)
Physician global assessment of disease activity (VAS 0-100)	−27.3 (−38.8)	−10.9 (−12.9)
Health Assessment Questionnaire (functional assessment) change	−0.38 (−21.3)	−0.07 (+1.8)
C reactive protein (mg/mL)	−19.5 (−42.8)	+3.0 (+0.4)
Erythrocyte sedimentation rate (mm/h)	−12 (−28.8)	−2.0 (−4.4)

Data are mean value (standard deviation) unless stated otherwise.

ACR20 is a composite measure defined as an improvement of 20% in the number of tender and swollen joints and a 20% improvement in three of the following five criteria: patient global assessment, physician global assessment, functional ability measure (usually the Health Assessment Questionnaire), visual analogue scale (VAS) on pain, and surrogate endpoints (erythrocyte sedimentation rate or C reactive protein. ACR50 and ACR70 are the same instruments as ACR20 but with improvement levels defined as 50% and 70%, respectively.

**Table 5:** Reporting of individual components of a composite measure (progression-free survival) in per protocol population, including recurrence or death. Table adapted from Parekh et al,^75^ with permission from Elsevier and Copyright Clearance Centre

Characteristic	Robotic cystectomy (n=150)	Open cystectomy (n=152)
Total events	49 (32.7)	50 (32.9)
Death from bladder cancer	28 (18.7)	32 (21.3)
Non-cancer death	10 (6.7)	11 (7.2)
Recurrence, alive at last contact	11 (7.3)	7 (4.6)
Any recurrence	39 (26.0)	39 (26.3)
Pure local recurrence (total)*	6 (4.0)	4 (2.6)
Cystectomy bed*	6 (4.0)	2 (1.3)
Pelvic lymphadenectomy template	0	1
Abdominal wall or port site	0	1
Distant recurrence (with or without local recurrence; total)	33 (22.0)	35 (23.0)
Lung	8	10
Liver	6	7
Bone	9	10
Extrapelvic lymph nodes	9	9
Peritoneal carcinomatosis	2	1
Adrenal	2	1
Colon	—	3
Small intestine	1	—
Kidney	1	—
Brain	—	1
Not specified	4	4
Secondary urothelial cancer (total)	1 (0.6)	3 (2.0)
Upper urinary tract	1	2
Urethra	0	1
Censored observations (total)	101 (67.3)	102 (67.1)
Censored within first two years	10 (6.7)	14 (9.2)
Under follow-up	9	8
Lost to follow-up	1	6

Data are number (%). Disease progression was defined by use of radiographical or pathological evidence of disease (surrogate endpoint), or death from disease (target outcome) according to Response Evaluation Criteria in Solid Tumours criteria version 1.1. Any documented recurrence or death from other causes was also regarded as progression.

* No significant difference was seen between the groups with respect to the incidence of total local recurrences (P=0.54) and cystectomy bed recurrences (P=0.17).

#### Explanation

A composite outcome comprises of two or more component outcomes (eg, the proportion of participants who had raised systolic blood pressure, experienced a non-fatal stroke, or died). Experience of any one of the components is considered as experience of the composite outcome.^25^ The considerations for using composite outcomes in trials are discussed in detail elsewhere and out of scope of this extension.^25^ ^76^ ^77^ ^78^

An audit of trials published in 2008-10 found that of 106 trials that used a composite outcome, 28% (n=30) included a surrogate endpoint as one of the components.^79^ Authors are encouraged to separately report the treatment effects on each component of a composite outcome. Reporting of this item applies to explicit composite outcomes (ie, “the primary outcome was a composite outcome [components a or b or c]”); and composite measures (table 4 and table 5). Composite measures and scales combine outcomes that are or include surrogate endpoint(s) such as disease or progression-free survival in cancer (disease recurrence or progression measured using tumour size or death)^80^; or clinical cure in infectious diseases, measured through clinician assessed response, and radiographical or microbiological criteria.^81^ ^82^ This item should preferably be included in the main text of the trial report rather than in supplementary files or appendices.

## Discussion

### Interpretation

#### CONSORT 2010 item 22 (extended)

Interpretation is consistent with results, balancing benefits, and harms, and considering other relevant evidence. 

Refer to CONSORT 2010.^4^ Reporting of the three subsequent CONSORT-Surrogate extension items can be done together and in any order.

#### CONSORT-Surrogate extension item 22.1

Interpretation of findings of the trial in the context of using a surrogate primary endpoint, including its known validity for intervention effects on the target outcome and the potential benefit-risk assessments of the tested intervention for participants.

#### CONSORT-Surrogate extension item 22.2

Comment on whether the trial design (including sample size and follow-up period), given the use of a surrogate endpoint, adequately captures the potential harms of the intervention being tested.

#### CONSORT-Surrogate extension item 22.3

State what the plans are to conduct subsequent analyses/studies to verify current findings on the target outcome(s).

### Examples of CONSORT-Surrogate item 22.1

#### Example 1

“With the detection rate for invasive breast cancer representing an early screening surrogate parameter, results from TOSYMA [trial name] point towards possible effects of digital breast tomosynthesis on long-term screening benefits. An absolute increase in the detection rate of invasive breast cancer for early tumour stages in the screening phase of TOSYMA, presumably indicating diagnostic improvements, might be expected to reduce the incidence of advanced breast cancers in screened populations and, thus, potentially exert positive effects on breast cancer mortality ^refs^. However, increased detection of small size cancers at screening without a reduction in the incidence of invasive interval cancers among screen negative women in the 2-year interval up to the subsequent screening examination would raise questions regarding the screening benefit and possible overdiagnosis ^refs^.^”^ 83

#### Example 2: Combining items 22.1 and 22.2

“If maintained in the long term as highlighted by the 3-year report of the Global SYMPLICITY Registry ^ref^ as well as the 12-month results of the RADIANCE-HTN SOLO study ^ref^, the average 9.0 mm Hg reduction in [the surrogate endpoint of] office systolic blood pressure we observed after renal denervation in patients with resistant hypertension who are at high risk of a cardiovascular event, ^ref^ is of a magnitude previously associated with a reduction in [target outcomes:] stroke, coronary heart disease, heart failure, and all-cause mortality for antihypertensive drug therapy ^ref^. A reduction in both cardiovascular and cerebrovascular events might also be expected if we confirm our previous observation in the RADIANCE-HTN SOLO trial of a reduced visit-to-visit variability in blood pressure after renal denervation ^ref^.”^10^ (We have added the words in square brackets and recommend their use when reporting the item.)

#### Explanation

This item recommends authors to consider the main limitations of the specific surrogate endpoints used and discuss the implications for interpreting the trial findings. Specifically, readers should be informed what the findings imply for the intervention effect observed on the surrogate endpoint and what it means for the target outcome drawing from current validity evidence (or lack of it); and potential overall intervention benefit-risk balance. In case of good predictive performance of surrogate validation models in the setting of interest, researchers should provide the predicted effect on the target outcome, together with a measure of uncertainty (eg, confidence intervals) and the actual prediction equation used. The following surrogate validation studies illustrate this level of reporting.^84^ ^85^ ^86^ Reporting of this item is important to inform how stakeholders use the trial findings to guide practice and policy.

Trials that collect data on both surrogate endpoint and target outcome with a more extended follow-up and larger sample sizes can be less speculative about the intervention benefit-risk balance. Irrespective of sample sizes and follow-up time, trial authors should report the treatment effects on the target outcome(s) when collected. Adequate reporting of other extension items on surrogate validity (item 6a.2) and potential harms (item 22.1) will enable adequate reporting of this item.

#### *Example of CONSORT-Surrogate item 22.2*

“Additional follow-up will be required to determine whether the blood pressure lowering effect of ultrasound renal denervation remains durable over time, especially when patients receive additional antihypertensive medications (particularly the aldosterone antagonist spironolactone) to control their blood pressure in both masked (2-6 months) and unmasked conditions (after 6 months) ^ref^. Although adverse events were infrequent, longer follow-up of this trial and more treated patients will be necessary to provide additional safety data.”^10^

#### Explanation

While trial treatment effects on a surrogate endpoint can indicate a potentially positive impact of an intervention, longer term trial follow-up or introduction of the intervention into routine practice could demonstrate the intervention to be harmful.^87^ In 1996, Fleming and DeMets described several examples where drugs had been approved on the basis of a positive treatment effect on a surrogate endpoint to be then shown to have overall harm to patients and the public.^88^ This example included suppression of arrhythmia (abnormal heart rhythm), where drugs to reduce arrhythmias (considered to be a surrogate endpoint for cardiovascular related mortality) were later found to increase mortality.^88^ More recent examples include a diabetes treatment (rosiglitazone) approved based on blood glucose reduction (a surrogate for serious diabetic complications, cardiovascular events, and death) that was later found to be associated with increased hospital admission for heart failure and increased heart attacks.^89^ Also, in the BELLINI trial, a drug (venetoclax) that improved progression-free survival (a surrogate for overall survival) in relapsed or refractory multiple myeloma patients was found to be associated with higher mortality.^90^

These harms could be due to various reasons, including unintended consequences of the intervention that are not mediated through the surrogate endpoint or known disease causal pathways; and intervention might not have a positive effect on the surrogate endpoint for the same people for whom the surrogate endpoint positively correlates with the target outcome.^87^ ^88^ When a surrogate endpoint is used as the primary outcome, we recommend collecting the target outcome as a secondary outcome, especially if it could inform potential harmful effects of the intervention and would override results based on the surrogate endpoint. For example, the BELLINI trial captured the harm of the intervention, leading to its early termination, because it used progression-free survival as a primary outcome but also collected overall survival as a secondary outcome.^90^ ^91^

#### *Examples of CONSORT-Surrogate item 22.3*

#### Example 1: Reporting subsequent analyses

“At a median follow-up of nearly 17 months, overall survival [target outcome] was not yet mature; however, fewer deaths occurred in the KdD [carfilzomib, dexamethasone, and daratumumab] group (19%) versus the Kd group [carfilzomib and dexamethasone] (23%), and a trend towards an overall survival benefit for KdD versus Kd was observed (appendix p 7). Overall survival will be reassessed in a subsequent pre-planned analysis.”^92^ (We have added the words in square brackets and recommend their use when reporting the item.)

#### Example 2: Combining items 22.1, 22.2, and 22.3 (reports ongoing study)

“This study is limited in that direct evaluation of the effect of vosoritide treatment on final adult height and how this relates to functionality, quality of life, and activities of daily living in people with achondroplasia cannot be evaluated at this time. In addition, whether treatment with vosoritide will ameliorate the medical complications associated with achondroplasia and decrease the need for surgical interventions is unknown.

“Concerns around these limitations are shared by some in the short-statured community, and their support groups, who consider that a treatment that only increases height [the surrogate endpoint] in achondroplasia is not a priority, and that the [target outcomes of] short term and long term health of individuals must also be enhanced. These perspectives are balanced by the views of some participants in this trial and their families, who agree that while better health is an important outcome, increased height in and of itself will facilitate better access to the environment, less discrimination, and higher self-esteem. To address these limitations, concerns, and unanswered questions, an ongoing, open-label, phase 3, extension study (ClinicalTrials.gov number, NCT03424018) will continue to evaluate the balance of benefits and harms of vosoritide until the patients reported in this study reach final adult height. This study will collect data regarding vosoritide therapy on wider health measures including quality of life, activities of daily living, and frequency and type of medical and surgical interventions compared with registry data of untreated children with achondroplasia. This long term study will also provide data on whether treatment of children with achondroplasia with vosoritide will result in a pubertal growth spurt, which appears to be absent in this condition ^ref^ and provide the opportunity to detect any harms associated with long term therapy.”^93^ (We have added the words in square brackets and recommend their use when reporting the item.)

#### Explanation

This item builds on the previous item to inform readers of subsequent analyses or studies to verify current findings (on observed benefit, lack of benefit, or harms) using a target outcome. These could include extended follow-up of the trial population to confirm the intervention effect on target outcome and evidence from surrogate endpoint validation studies. A survey of cardiovascular trials using surrogate endpoints as primary outcomes and published in three high impact journals between 1990 and 2011 found that only 27% had subsequent trials to verify findings using a target outcome.^94^ In cancer, a retrospective analysis of drug approvals by the US Food and Drug Administration (FDA) found that 56% of accelerated approvals and 37% traditional approvals were not supported by strong surrogate validation evidence.^95^ Nevertheless, only 45% of the approvals had subsequent analysis on the target outcome of overall survival.^95^ Lack of subsequent studies to verify the effect could extend beyond cardiovascular diseases, cancer, and drug related interventions, and could lead to the continued use of interventions that have no benefit.^23^

We acknowledge that the conduct of subsequent trial analyses or additional studies depends on several factors, including feasibility and availability of research funding. Furthermore, plans to conduct such future analyses studies could change over time. Nevertheless, we recommend that authors are transparent in reporting this item—that is, explicit statement of no plans (with justification), description of plans (including planned follow-up beyond study period, planned or confirmatory target outcome trial in progress), or description of initial plans that have changed.

## Other information

### New items

#### CONSORT-Surrogate extension item 26.1

State whether and how trial participants were engaged and informed before enrolment that the trial was designed to evaluate an intervention’s effect using a surrogate endpoint.

#### CONSORT-Surrogate extension item 26.2

If surrogate endpoint and target outcome data were collected in the trial, state the open access arrangements for the data for future secondary research.

### Example of CONSORT-Surrogate item 26.1

“All participants [received] adequate information about the nature, purpose, possible risks, and benefits of the trial [given the use of a surrogate endpoint as the primary outcome], and alternative therapeutic choices using an informed consent protocol approved by the IRB [institutional review board]. All participants [were] given ample time and opportunity to ask questions and consider participation in the trial.”^96^ (This example did not implement the item but has been used to show how the item can be reported using the words in square brackets. It was also taken from a protocol and has been modified to show past tense.)

#### Explanation

Public engagement (also known as community engagement) is listening to, interacting with, and connecting with members of the public to share research activity or benefits, discuss relevant issues (such as ethics), or obtain input on preliminary research ideas.^97^ Patient and public involvement is focused on a specific study and involves conduct of research with or by members of the public (rather than “for,” “to,” or “about” members of the public).^97^ Public engagement is vital for both planning and conduct of trials but also translation of trial findings, and greater benefit for trial participants and the public.^98^ ^99^ Public engagement and informed consent are mutually supportive issues with the same goal: maximising social value of research conducted in a respectful manner.^100^ ^101^ ^102^ Informed consent is a legal and ethical requirement of research involving human participants before their enter the study.^103^ ^104^ It involves adequately informing participants of trial details including the anticipated benefits and potential risks of participation.^103^ ^105^ Therefore, for trials using surrogate endpoints as primary outcomes, informed consent provides an avenue to engage trial participants on the use of surrogate endpoints and their risks and benefits or ideally to continue ongoing engagement. However, evidence from early phase trials (many of which might rely on surrogate endpoints) suggests that participant risk-benefit communication is suboptimal. A survey of 172 early phase trials’ informed consent documents found that only 45% specified the outcome of mentioned health benefits, and only 63% mentioned the likelihood of health risks of which only half were specific on whether risks would be due to research procedures or potentially beneficial interventions.^105^

Informing trial participants that the study used a surrogate endpoint (and related limitations) is critical to informed consent.^106^ We have discussed this item in detail in the SPIRIT-Surrogate extension, including suggestions on implementing it. Authors should justify trials that do not implement the item.

### Examples of CONSORT-Surrogate item 26.2

#### Example 1: Data available on request

**“**Data Sharing Statement: The complete deidentified patient data set will be made available upon publication to researchers whose proposed use of the data has been approved. Requests should be sent to ctu.beatlupus@ucl.ac.uk.”^107^ (The trial’s primary outcome was the surrogate endpoint of levels of anti-double stranded DNA antibodies in serum IgG, and disease flares were the target outcome which was a secondary outcome.)

#### Example 2: Data available via an intermediary

“Janssen has an agreement with the Yale Open Data Access (YODA) Project to serve as the independent review panel for the evaluation of requests for clinical study reports and participant-level data from investigators and physicians for scientific research that will advance medical knowledge and public health. Data will be made available following publication and approval by YODA of any formal requests with a defined analysis plan. For more information on this process or to make a request, please visit the Yoda Project site at https://yoda.yale.edu. The data sharing policy of Janssen Pharmaceutical Companies of Johnson & Johnson is available at https://www.janssen.com/clinical-trials/transparency.”^108^ (This phase 1 trial had safety as the primary outcome but is an example of the deposit of data in an intermediary.)

#### Explanation

We have already highlighted the importance of collecting target outcome data when a surrogate endpoint is used as a primary outcome: it can allow for surrogate endpoint validation and can contribute to monitoring intervention harms. Therefore, we encourage trial teams to collect target outcomes as secondary outcomes. A key challenge of undertaking surrogate validation studies is limited access of individual participant data from completed studies.^109^ Therefore, sharing surrogate endpoint and target outcome data, when collected, allows leveraging the trial dataset to advance the surrogate validation field.

Adequate reporting of this item (ie, statements that data will be available) is not enough: trial investigator teams should be genuinely committed to sharing their datasets. Recent surveys of published trials found that access to individual patient level data was overwhelmingly low (<25%) despite most trial authors having declared an intention to share the data.^110^ ^111^ There could be challenges to data sharing, including risk of loss of participant confidentiality, perceived risk of inappropriate use of data, and competition from peers with access to the data.^110^ ^112^ Consequently, when data sharing is impossible or only possible for part of the data; authors should be explicit about it with a justification.

## Conclusion

Trials using surrogate endpoints need better, more transparent reporting. The CONSORT-Surrogate extension provides the minimum reporting requirements for trial reports and publications that have used surrogate endpoints as primary outcomes. Proper application of CONSORT-Surrogate should improve reporting of such trials, aiding interpretation of findings to inform practice and policy. The extension should be used along with the main CONSORT 2010 reporting guideline.

The CONSORT-Surrogate extension can contribute to reduction of research waste.^3^ Nevertheless, while many journals endorse using the main CONSORT checklists, very few endorse using extensions.^1^ We therefore call on all stakeholders (including funders, journal editors, and reviewers) to encourage use of the CONSORT-Surrogate extension. However, we acknowledge that use of the extension does not rule out other sources of research waste, including wrong choice of research question, biases, or poor design.^2^ Specifically, trial teams and readers should note that bias in surrogate endpoint measurement contributes to poor prediction of intervention effects on target outcomes.^16^ Finally, adequate reporting of all items in this extension does not preclude trial investigator teams and the wider scientific community from directly assessing and reporting intervention effects on target outcomes, whenever possible.

## Data Availability

Additional data are available through request from the corresponding author. After publication of all project’s manuscripts, data will be deposited in the UK Data archive, and will be accessed through their standard end user licence (this would require users to login to the UK Data Service).

## Web extra

Extra material supplied by authors

Web appendix 1: Supplementary material

Web appendix 2: Acknowledgments

Web appendix 3: Combined CONSORT-Surrogate extension checklist

## Notes

Contributors: AMM, RST, and OC are joint first authors. PD, CJW, AEY, RST, and OC were involved in funding acquisition. AMM, PD, DS, CJW, AEY, RST, and OC were involved in study conception and design. AMM, PD, DS, CJW, AEY, JB, RST, OC, NJB, SB, A-WC, GSC, DD, MOf, MOu, and JSR contributed to the methodology. NJB, SB, A-WC, GSC, DD, MOf, MOu, and JSR supervised the project. AMM, RST, and OC curated the data and conducted formal analysis. AMM, RST, OC, SM, DS, RH, and AW were responsible for the first draft of the manuscript. All authors critically reviewed the first draft and approved the final version. AMM, RST, and OC are the guarantors. The corresponding author confirms that all listed authors meet authorship criteria and that no others meeting the criteria have been omitted.

Transparency: The guarantors affirm that this manuscript is an honest, accurate, and transparent account of the study being reported; that no important aspects of the study have been omitted; and that any discrepancies from the study as planned (and, if relevant, registered) have been explained.

Patient and public involvement: Four patient and public involvement (PPI) partners (DS, SM, RH, AW) were consensus meeting delegates and are coauthors of this extension. DS has been a member of the project team and the project PPI lead. Nineteen PPI partners participated in the e-Delphi survey.

Dissemination to participants and related patient and public communities: The SPIRIT-Surrogate and CONSORT-Surrogate extensions will be disseminated to the public through press releases, presentations at conferences, video tutorials, and plain language summaries posted on websites and social media.

Provenance and peer review: Not commissioned; externally peer reviewed.
