# Supplementary material for: Reporting of surrogate endpoints in randomised controlled trial reports (CONSORT-Surrogate): extension checklist with explanation and elaboration
Source: BMJ. 2024 Jul 9;386:e078524. doi: 10.1136/bmj-2023-078524 (PMC11231881; doi:10.1136/bmj-2023-078524)
Supplement: Supplementary file 1 — Web appendix 1: Supplementary material [file mana078524.ww1.pdf]

Supplementary material

Reporting of surrogate endpoints in randomised controlled trial reports: The CONSORT-Surrogate extension checklist with explanation and elaboration

Table of Contents

Methodological consideration in design and reporting of surrogate endpoints in trials. 2

Table A.1: Methodological considerations when designing and reporting trials using surrogate endpoints. 2

Characteristics of Delphi survey participants and Consensus Meeting delegates 4

Table A.2: Country of residence of participants of Delphi survey and consensus meeting delegates 4

Table A.3: Primary and secondary roles of Delphi, consensus meeting, and definition survey participants 6

Table A.4: Research and disease areas of expertise of Delphi, consensus meeting, and definition survey participants 8

Table A.5: Employment sector and experience characteristics of Delphi and consensus meeting participants 10

Delphi Round 2 and Consensus Meeting results 11

Table A.6: Rating of checklist items and consensus decision in Round 2 11

Table A.7: Summary of discussion and voting on items in the consensus meeting. 13

Methodological consideration in design and reporting of surrogate endpoints in trials.

Table A.1: Methodological considerations when designing and reporting trials using surrogate endpoints.

Consideration	Description	Why it is important	Mitigating strategies
Definition	Defining when a measure is considered a surrogate endpoint can be challenging	A mismatch between stakeholders on what is considered a surrogate endpoint could lower the value of findings in informing practice and policy	▪ Careful consideration of different criteria and definitions to determine a trial measure is a surrogate endpoint[1]<br>▪ Stakeholders (including trial participants, trial investigators, clinicians, regulators, and payers) should engage to determine when a measure should be a surrogate for a target outcome.
Explicit mention	Use of a surrogate endpoint should be explicitly mentioned in a trial report	Readers should know when a trial investigator team considered their primary outcome as a surrogate without the need of implying from report	▪ Trial investigators should explicitly report that the primary outcome was a surrogate – see items 1b, 2.1 of this extension
Validation	Where possible, validated surrogate endpoints should be used in trials.	Use of a surrogate that is not valid (i.e., not accurately predictive of intervention effect on the target outcome) will likely lead to inaccurate estimation of true intervention effects	▪ Justification of using the surrogate outcome should be made using validation evidence – see item 6a.2 of this extension.<br>▪ Consideration of collecting target outcomes as secondary outcomes and conducting subsequent studies/analyses to verify surrogate findings – see item 22.3 of this extension
Sample size informed by surrogate validity	In calculating the sample size, the minimum effect on a meaningful change in the surrogate endpoint (estimated from surrogate validation) should be used.	Failure to consider a meaningful intervention effect size on the surrogate endpoint in determining sample sizes will likely lead to the imprecision of the predictive effect on the intervention target outcome.	▪ Surrogate validity metrics used to determine sample size whenever possible – see item 7a.1 of this extension
Surrogate endpoint in a composite outcome	Sometimes a surrogate endpoint can contribute as part of composite outcome	Overall conclusions on the composite could be driven by surrogate endpoint rather than other contributing target outcomes.	▪ Authors report intervention effects of individual components of the composite outcome individually – see item 17a.1 of this extension
Intervention harms	Surrogate endpoints are typically associated with efficacy/effectiveness and, as a	Harms are important for an intervention’s evaluation, approval, and use.	▪ Trial investigator teams should consider monitoring harms such as extended follow-up and collecting target outcomes as secondary outcomes

	result, fail to capture the potential harms of an intervention.		and explicitly mention it – see item 22.1 of this extension.<br>▪ Communication of uncertainty in the likelihood of harms
Interpretation of findings	Readers should know what trial findings imply when a surrogate endpoint is used so they: can weigh up the potential balance in intervention benefits and risks	Use of surrogate endpoints may fail to accurately reflect intervention and estimate efficacy/effectiveness and harms.	▪ Findings should be interpreted carefully (including consideration of surrogate endpoint validity) as to the overall impact of the intervention – see item 22.2 of this extension
Public engagement	As part of the public engagement and consent process, trial participants should be informed on the use of surrogate endpoint	Trials participants should understand the rationale of the use and the uncertainty associated with a surrogate endpoint primary outcome.	▪ Trial investigating teams should consider fully informing and engaging with participants when a surrogate endpoint is used and report on the level of these engagements – see item 26.1 of this extension
Data for surrogate validation	Surrogate validation requires data on the intervention effect on both the surrogate endpoint and the target outcome	One of the challenges of surrogate validation is limited data availability and limited ability to extrapolate validity from one (intervention or trial population) context to another	▪ Trial investigator teams should consider collection both surrogate endpoint and target outcome data.<br>▪ When this data is collected it should be made available for secondary research, such as surrogate validation – see item 26.2 of this extension

Characteristics of Delphi survey participants and Consensus Meeting delegates

Table A.2: Country of residence of participants of Delphi survey and consensus meeting delegates

Country	Registered and eligible N=212	Round 1 respondents N=195	Round 2 respondents N=176	Consensus meeting delegates N=33
	n (%)	*n (%)*	*n (%)*	*n (%)*
**Australia**	8 (3.8)	7 (3.6)	5 (2.8)	1 (3.0)
**Austria**	1 (0.5)	0 (0)	0 (0)	0 (0)
**Belgium**	3 (1.4)	3 (1.5)	3 (1.7)	0 (0)
**Brazil**	2 (0.9)	2 (1.0)	1 (0.6)	1 (3.0)
**Canada**	14 (6.6)	14 (7.2)	12 (6.8)	2 (6.1)
**Chile**	1 (0.5)	1 (0.5)	1 (0.6)	0 (0)
**Croatia**	1 (0.5)	1 (0.5)	1 (0.6)	0 (0)
**Denmark**	6 (2.8)	6 (3.1)	6 (3.4)	2 (6.1)
**Finland**	1 (0.5	1 (0.5)	1 (0.6)	0 (0)
**France**	5 (2.4)	5 (2.6)	5 (2.8)	1 (3.0)
**Germany**	6 (2.8)	5 (2.6)	5 (2.8)	1 (3.0)
**Greece**	2 (0.9)	2 (1.0)	2 (1.1)	0 (0)
**Hungary**	1 (0.9)	0 (0)	1 (0.6)	0 (0)
**India**	3 (1.4)	2 (1.0)	1 (0.6)	0 (0)
**Iran**	1 (0.9)	1 (0.5)	1 (0.6)	1 (3.0)
**Ireland**	5 (2.4)	5 (2.6)	5 (2.8)	1 (3.0)
**Israel**	1 (0.9)	1 (0.5)	1 (0.6)	0 (0)
**Italy**	20 (9.4)	18 (9.2)	18 (10.2)	3 (6.1)
**Malaysia**	1 (0.5)	1 (0.5)	1 (0.6)	0 (0)
**Netherlands**	4 (1.9)	4 (2.1)	4 (2.3)	0 (0)

**New Zealand**	4 (1.9)	3 (1.5)	3 (2.3)	0 (0)
**Nigeria**	1 (0.5)	1 (0.5)	0 (0)	0 (0)
**Philippines**	3 (1.4)	3 (1.5)	3 (1.7)	0 (0)
**South Africa**	1 (0.5)	0 (0)	0 (0)	0 (0)
**Spain**	4 (1.9)	4 (2.1)	3 (1.7)	0 (0)
**Sweden**	2 (0.9)	2 (1.0)	2 (1.1)	0 (0)
**Switzerland**	6 (2.8)	6 (3.1)	6 (3.4)	1 (3.0)
**Tanzania**	1 (0.5)	1 (0.5)	0 (0)	0 (0)
**Thailand**	1 (0.5)	1 (0.5)	1 (0.6)	0 (0)
**Turkey**	1 (0.5)	1 (0.5)	1 (0.6)	0 (0)
**UK**	69 (32.5)	65 (33.3)	61 (34.7)	13 (39.4)
**USA**	33 (15.6)	29 (14.9)	22 (12.5)	5 (15.2)

Table A.3: Primary and secondary roles of Delphi, consensus meeting, and definition survey participants

Role	Registered & eligible N=212		Round 1 respondents N=195		Round 2 respondents N=176		Consensus meeting delegates N=33	
	Primary role	***Secondary role****	***Primary role***	***Secondary role****	***Primary role***	***Secondary role****	***Primary role***	***Secondary role****
	n (%)	***n (%)***	***n (%)***	***n (%)***	***n (%)***	***n (%)***	***n (%)***	***n (%)***
**Clinician/Health and allied health professional**	38 (17.9)	49 (23.1)	32 (16.4)	45 (23.1)	31 (17.6)	41 (23.3)	6 (18.2)	9 (27.3)
**Trial methodologist**	33 (15.6)	41 (19.3)	32 (16.4)	38 (19.5)	30 (17.0)	36 (20.5)	7 (21.2)	9 (27.3)
**Trial investigator**	30 (14.2)	42 (19.8)	27 (13.8)	39 (20.0)	21 (11.9)	38 (21.6)	0 (0)	14 (42.4)
**Statistician**	29 (13.7)	15 (7.1)	27 (13.8)	15 (7.7)	26 (14.8)	13 (7.4)	6 (18.2)	4 (12.1)
**Epidemiologist**	12 (5.7)	18 (8.5)	12 (6.2)	16 (8.2)	12 (6.8)	16 (9.1)	2 (6.1)	3 (9.1)
**HTA expert/Health economist**	14 (6.6)	8 (3.8)	12 (6.2)	8 (4.1)	12 (6.8)	8 (4.5)	1 (3.0)	4 (12.1)
**Journal editor/associate editor/editorial board member**	8 (3.8)	25 (11.8)	8 (4.1)	23 (11.8)	7 (4.0)	21 (11.9)	5 (15.1)	10 (30.3)
**PPI partner**	15 (7.1)	4 (1.9)	15 (7.7)	4 (2.1)	12 (6.8)	7 (4.0)	4 (12.1)	0 (0)
**Surrogate content expert**	6 (2.8)	8 (3.8)	6 (3.1)	7 (3.6)	6 (3.4)	8 (4.5)	2 (6.1)	3 (9.1)
**Regulatory assessor**	4 (1.9)	5 (2.4)	4 (2.1)	4 (2.1)	3 (1.7)	4 (2.3)	0 (0)	1 (3.0)
**Research ethics committee member**	4 (1.9)	10 (4.7)	4 (2.1)	7 (3.6)	3 (1.7)	6 (3.4)	0 (0)	0 (0)
**Clinical guideline/core outcome set developer**	3 (1.4)	19 (9.0)	2 (1.0)	19 (9.7)	1 (0.6)	19 (10.8)	0 (0)	5 (15.1)
**Trial manager**	6 (2.8)	8 (3.8)	6 (3.1)	7 (3.6)	5 (2.8)	7 (4.0)	0 (0)	2 (6.1)

**Research funding board/panel member**	1 (0.5)	19 (9.0)	1 (0.5)	16 (8.2)	1 (0.6)	18 (10.2)	0 (0)	5 (15.1)
**Other**	9 (4.2)	12 (5.7)	7 (3.6)	10 (5.1)	6 (3.4)	5 (28.4)	0 (0)	1 (3.0)
**Not applicable**		19 (9.0)		18 (9.2)		16 (9.1)	0 (0)	3 (9.1)
***Overlapping**								

Table A.4: Research and disease areas of expertise of Delphi, consensus meeting, and definition survey participants

Research/disease area	Registered & eligible N=212	Round 1 respondents N=195	Round 2 respondents N=176	Consensus meeting delegates N=33
	n (%)	*n (%)*	*n (%)*	*n (%)*
1. **Ageing**	1 (0.5)	1 (0.5)	1 (0.6)	0 (0)
2. **Anaesthesia, perioperative medicine, and critical care**	3 (1.4)	3 (1.5)	1 (0.6)	1 (3.0)
3. **Cancer**	43 (20.3)	41 (21.0)	35 (19.9)	1 (3.0)
4. **Cardiovascular diseases**	13 (6.1)	13 (6.7)	10 (5.7)	5 (15.1)
5. **Dermatology**	1 (0.5)	1 (0.5)	1 (0.6)	0 (0)
6. **Ear, Nose, and Throat**	4 (1.9)	4 (2.1)	4 (2.3)	0 (0)
7. **Gastroenterology**	3 (1.4)	3 (1.5)	3 (1.7)	1 (3.0)
8. **Global Health**	2 (0.9)	2 (1.0)	2 (1.1)	0 (0)
9. **Haematology**	2 (0.9)	2 (1.0)	2 (1.1)	0 (0)
10. **Hepatology**	1 (0.5)	1 (0.5)	1 (0.6)	0 (0)
11. **Infectious diseases**	10 (4.7)	10 (5.1)	9 (5.1)	4 (12.1)
12. **Mental health**	4 (1.9)	2 (1.0)	3 (1.7)	1 (3.0)
13. **Metabolic and endocrine**	4 (1.9)	3 (1.5)	4 (2.3)	0 (0)
14. **Musculoskeletal**	18 (8.5)	16 (8.2)	14 (8.0)	3 (9.1)
15. **Neuro-progressive and Dementia**	4 (1.9)	3 (1.5)	3 (1.7)	0 (0)
16. **Ophthalmology**	1 (0.5)	1 (0.5)	1 (0.6)	0 (0)
17. **Oral and dental**	1 (0.5)	1 (0.5)	1 (0.6)	0 (0)
18. **Paediatrics**	5 (2.4)	5 (2.6)	5 (2.8)	1 (3.0)
19. **Pain**	1 (0.5)	1 (0.5)	1 (0.6)	0 (0)
20. **Primary care**	4 (1.9)	4 (2.1)	4 (2.3)	1 (3.0)
21. **Public health/health promotion**	8 (3.8)	6 (3.1)	5 (2.8)	1 (3.0)
22. **Rehabilitation and physical therapy**	6 (2.8)	6 (3.1)	5 (2.8)	2 (6.1)
23. **Renal medicine**	3 (1.4)	3 (1.5)	3 (1.7)	0 (0)
24. **Reproductive and childbirth**	3 (1.4)	3 (1.5)	2 (1.1)	1 (3.0)
25. **Respiratory**	5 (2.4)	4 (2.1)	4 (2.3)	0 (0)

26. **Trauma and Emergencies**	1 (0.5)	1 (0.5)	1 (0.6)	0 (0)
27. **Other**	15 (7.1)	12 (6.2)	12 (6.8)	2 (6.1)
28. **Not applicable**	24 (11.3)	24 (12.3)	22 (12.5)	5 (15.1)
29. **Did not answer**	22 (10.4)	19 (9.7)	17 (9.7)	4 (12.1)

Table A.5: Employment sector and experience characteristics of Delphi and consensus meeting participants

Characteristics	Registered & eligible N=212	Round 1 respondents N=195	Round 2 respondents N=176	Consensus meeting delegates N=33
**Employment sector*- n (%)**				
**Academia**	139 (65.6)	126 (64.6)	113 (64.2)	26 (78.8)
**Healthcare facility/hospital**	65 (30.7)	59 (30.3)	54 (30.7)	10 (30.3)
**Healthcare industry/Consultancy**	26 (12.3)	22 (11.3)	20 (11.4)	3 (9.1)
**Regulatory or payer agency**	9 (4.2)	9 (4.6)	7 (4.0)	2 (6.1)
**Other**	16 (7.5)	16 (8.2)	16 (9.1)	0 (0)
**Not employed**	12 (5.7)	12 (6.2)	24 (13.6)	2 (6.1)
**Surrogate endpoint trials related experience or expertise***				
**Design, conduct, and reporting of trials**	145 (68.4)	135 (69.2)	121 (68.8)	24 (72.3)
**Publishing methodological research outputs or surrogate endpoint content**	110 (51.9)	104 (53.3)	95 (54.0)	14 (42.4)
**Review/synthesis of evidence from trials**	108 (50.9)	99 (50.8)	92 (52.3)	21 (63.7)
**Peer review of trials**	95 (44.8)	90 (46.2)	89 (50.6)	24 (72.7)
**Peer review or commissioning of grants or funding applications**	62 (29.2)	59 (30.3)	52 (29.5)	17 (51.5)
**Ethical review of trials**	27 (12.7)	24 (12.3)	21 (11.9)	1 (3.0)
**Regulatory assessment or cost evaluation**	32 (15.1)	29 (14.9)	27 (15.3)	7 (21.2)
**End-user of intervention(s) approved based on surrogacy evidence**	17 (8.0)	16 (8.2)	15 (8.5)	3 (9.1)
**Implementer of intervention(s) approved based on surrogacy evidence**	13 (6.1)	10 (5.1)	10 (5.7)	1 (3.0)
**Other**	12 (5.7)	10 (5.1)	5 (3.1)	0 (0)
**Not applicable**	12 (5.7)	10 (5.1)	10 (5.7)	1 (3.0)
**Years of experience – Median (IQR)**	15 (8, 20)	15 (8, 20)	15 (8, 20)	17 (10, 20)
***Overlapping**				

Delphi Round 2 and Consensus Meeting results

Table A.6: Rating of checklist items and consensus decision in Round 2

Item	N	Median (IQR)	% on the rating scale			Consensus decision
			1-3	4-6	7-9	
SPIRIT 1 [M]: State that primary outcome(s) is considered a surrogate endpoint	176	9 (8, 9)	1.7	6.8	91.5	Consensus in Round 1
CONSORT 1 [M]: State that primary outcome(s) is considered a surrogate endpoint	176	9 (8, 9)	1.7	7.4	90.9	Consensus in Round 1
SPIRIT 2 [M]: State the participant/patient relevant final outcome(s) that the surrogate endpoint is substituting and predicting for	175	9 (7, 9)	1.1	7.4	91.4	Consensus in Round 1
CONSORT 2 [M]: State the participant/patient relevant final outcome(s) that the surrogate endpoint is substituting and predicting for	175	9 (7, 9)	1.1	8.0	90.9	Consensus in Round 1
SPIRIT 3 [M]: State the practical reason(s) for using a surrogate endpoint as a primary outcome	176	8 (7, 9)	0.6	10.8	88.6	Consensus in Round 1
CONSORT 3 [M]: State the practical reason(s) for using a surrogate endpoint as a primary outcome	176	8 (7, 9)	0.6	13.1	86.4	Consensus in Round 1
SPIRIT 4 [M]: Justification for selected surrogate: Evidence of validation	176	9 (8, 9)	0.0	4.5	95.5	Consensus in Round 1
CONSORT 4 [M]: Justification for selected surrogate: Evidence of validation	176	9 (7, 9)	0.6	6.8	92.6	Consensus in Round 1
SPIRIT 5 [M]: Justification for selected surrogate: Evidence of being specific to the setting used, e.g., intervention, disease, population	176	8 (7, 9)	0.6	10.8	88.6	Consensus in Round 1
CONSORT 5 [M]: Justification for selected surrogate: Evidence of being specific to the setting used, e.g., intervention, disease, population	176	8 (7, 9)	0.6	10.8	88.6	Consensus in Round 1
[Suggested Item] SPIRIT/CONSORT: State what other surrogate endpoints were considered and why the current one(s) were chosen over those.	172	6 (5, 8)	10.5	45.9	43.6	Discussion and vote in the consensus meeting

**SPIRIT 6 [M]: Clarify if the sample size calculation is explicitly informed by statistical metrics of surrogate validity	169	7 (7, 9)	1.8	19.5	78.7	Consensus in Round 2
**CONSORT 6 [M]: Clarify if the sample size calculation was explicitly informed by statistical metrics of surrogate validity	169	7 (7, 9)	1.8	21.3	76.9	Consensus in Round 2
**SPIRIT 7 [N]: State if trial participants will be informed before enrolment that the trial was designed to evaluate an interventions effect using a surrogate endpoint	174	6 (5, 7.8)	12.1	47.1	40.8	Discussion and vote in the consensus meeting
**CONSORT 7 [N]: State if trial participants were informed before enrolment that the trial was designed to evaluate an interventions effect using a surrogate endpoint	174	6 (4.3, 7)	12.1	52.3	35.6	
CONSORT 8 [M]: If the primary outcome is a composite outcome that includes a surrogate endpoint, report the intervention effect on all components	173	9 (7, 9)	0.6	6.4	93.1	Consensus in Round 1
SPIRIT 8 [M]: Comment on whether the trial sample size and follow-up period are sufficient to adequately capture the potential harms of the intervention being tested	175	8 (7, 8.5)	1.7	10.3	88.0	Consensus in Round 1
CONSORT 9 [M]: Comment on whether the trial sample size and follow-up period are sufficient to adequately capture the potential harms of the intervention being tested	175	8 (7, 9)	1.1	12.0	86.9	Consensus in Round 1
SPIRIT 9 [N]: State if there are explicit plans to extend follow-up or conduct subsequent analyses/studies to verify the benefit of current findings on the patient relevant final outcome	176	6 (6, 7)	3.4	50.0	46.6	Discussion and vote in consensus meeting
CONSORT 10 [N]: State if there are explicit plans to extend follow-up or conduct subsequent analyses/studies to verify the benefit of current findings on the patient relevant final outcome	176	6 (6, 7)	3.4	47.2	49.4	
**CONSORT 11 [M]: Provide an estimate (with a measure of uncertainty) of the predicted effect of patient relevant final outcome based on the observed effect on the surrogate endpoint; and if not possible, then a qualitative assessment	173	7 (6, 8)	4.0	38.2	57.8	Discussion and vote in consensus meeting
CONSORT 12 [M]: Interpretation of findings of the trial in the context of using a surrogate primary endpoint, including its known validity and the potential benefit-risk ratio of the tested intervention for participants	174	8 (7, 9)	1.7	13.2	85.1	Consensus in Round 1
**CONSORT 13 [M]: If surrogate endpoint and patient relevant final outcome data were collected in the trial; state the open access arrangements for the data for future secondary research, including the validation of the surrogate endpoint	172	7 (6, 8)	1.7	27.9	70.3	Consensus in Round 2

IQR – Interquartile Range
*Some items have been merged or changed in their wording following the consensus meeting and write up

Table A.7: Summary of discussion and voting on items in the consensus meeting.

Item	Summary of discussion points	Voting outcome	Consensus decision for extensions
State what other surrogate endpoints were considered and why the current one(s) were chosen over those *(For SPIRIT and CONSORT)*	▪ Reporting the item is part of transparency as surrogate endpoints, especially those less well-established, can be cherry-picked.<br>▪ Allows for identifying strengths and weaknesses of selected surrogate endpoints.<br>▪ Consideration of not including the item as justification of selected surrogate endpoints already covered by the item on validation.<br>▪ There can be strength in repetition, and authors should be clear on alternative surrogate endpoints considered.<br>▪ Consider inclusion in SPIRIT and not in CONSORT to limit duplication.	**SPIRIT (n=27)**<br>Include= 70%<br>Exclude= 22%<br>Unsure= 7%<br>**CONSORT (n=29)**<br>Include= 17%<br>Exclude= 78%<br>Unsure= 7%	Include in SPIRIT<br><br>Exclude in CONSORT
State if trial participants will be/were informed before enrolment that the trial was designed to evaluate an intervention's effect using a surrogate endpoint. *(For SPIRIT and CONSORT)*	▪ Guarantees actual informed consent before participation in a trial.<br>▪ Feasibility: Item can be implemented if the information is clearly explained to participants.<br>▪ Allows for participants to understand from the onset why trials showing positive effects may not result in access or approval of an intervention.<br>▪ Important for items to be captured in informed consent for trials using surrogate endpoints as primary outcomes, irrespective of whether target outcomes are collected as secondary outcomes.<br>▪ When benefit is measured using surrogate endpoints, but harms measured on direct outcomes, it changes the quantitative assessment of benefit versus harms; and changes as more data on surrogate endpoints accumulates.<br>▪ Difficult to access informed consent forms for secondary research therefore, trial authors reporting this item is critical.<br>▪ Should the level of evidence (validation) supporting the use of the surrogate endpoint should be communicated? Some delegates felt that this should be avoided unless there is a qualification from a regulatory agency.<br>▪ Implementing the item has a risk of lowering participation.<br>▪ Should trial teams consider informing participants if there is follow-up to measure the target outcomes after the use of a surrogate in the current trial?<br>▪ Consider having the item only in SPIRIT.<br>▪ Some trials have no published protocols, and most readers may only read the trial reports hence need to have items in both SPIRIT and CONSORT.	**SPIRIT (n=28)**<br>Include= 96%<br>Exclude= 4%<br>Unsure= 0%<br>**CONSORT (n=27)**<br>Include= 74%<br>Exclude= 19%<br>Unsure= 7%	Include in SPIRIT<br><br>Include in CONSORT

	▪ Risk of tokenism in implementing this item: need for genuine effort by trial teams to inform participants.<br>▪ Novel item hence guidance needed for implementation		
State if there are explicit plans to conduct subsequent analyses/studies to verify the benefit of current findings on the patient-relevant final outcome. *(For SPIRIT and CONSORT)*	▪ Part of transparency in reporting of trials<br>▪ Trials with follow-up built in are completed earlier and result to faster regulatory action on drugs, https://jamanetwork.com/journals/jama/fullarticle/2801050<br>▪ Some trials cross over controls to receive intervention hence makes it harder to follow up randomised groups.<br>▪ Consider following participants for the target outcome within the trial, if possible, such as in infectious diseases. Furthermore, crucial to collect and report the target outcome even when the study is not powered for it. Target outcomes should be included as secondary outcomes even when effect sizes are unknown.<br>▪ Important to have this information for meta-analysis and surrogate validation studies.<br>▪ This item is dependent on resources (time and funding) and context as for some trials/research areas, surrogate endpoints are used as it is impossible or less practical to use/collect the target outcome.<br>▪ Need for modification of wording to reflect "what plans"	**SPIRIT (n=30)**<br>Include= 93%<br>Exclude= 3%<br>Unsure= 3%<br>**CONSORT (n=30)**<br>Include= 83%<br>Exclude= 10%<br>Unsure= 7%	Include in SPIRIT<br><br>Include in CONSORT
Provide an estimate (with a measure of uncertainty) of the predicted effect of patient-relevant final outcome based on the observed effect on the surrogate endpoint; and if not possible, then a qualitative assessment *(For CONSORT)*	▪ Participants/patients would want to know how much effect on surrogate endpoints means on the target outcome.<br>▪ Difficult to provide an estimate of predicated effect, but qualitative assessment should always be there.<br>▪ Risk of introducing spin – misleading reporting, interpretation, or extrapolation of findings<br>▪ Can only be implemented in validated surrogate endpoints, and most disease/research areas may not have data to support estimations. Furthermore, estimates can be analysed for trials that collect both surrogate and target outcome data.<br>▪ Consider discussing the item in the Explanation and Elaboration	**CONSORT (n=30)**<br>Include=13%<br>Exclude=73%<br>Unsure=13%	Exclude in CONSORT

1. Ciani O, Manyara AM, Davies P, et al. A framework for the definition and interpretation of the use of surrogate endpoints in interventional trials. *eClinicalMedicine* 2023;65:102283. doi: https://doi.org/10.1016/j.eclinm.2023.102283
