# Supplementary material for: Reporting of surrogate endpoints in randomised controlled trial reports (CONSORT-Surrogate): extension checklist with explanation and elaboration
Source: BMJ. 2024 Jul 9;386:e078524. doi: 10.1136/bmj-2023-078524 (PMC11231881; doi:10.1136/bmj-2023-078524)
Supplement: Supplementary file 3 — Web appendix 3: Combined CONSORT-Surrogate extension checklist [file mana078524.ww3.pdf]

SPIRIT | CONSORT SURROGATE
Reporting Guidelines for Surrogate Endpoints

The combined CONSORT- Surrogate extension checklist

Section/Topic	Item No	CONSORT- Surrogate items	Reported on page No
	**Title and abstract**		
	1a	Identification as a randomised trial in the title	
	1b	Structured summary of trial design, methods, results, and conclusions (for specific guidance, see CONSORT for abstracts)	
	1b.1	State a) that the primary outcome is a surrogate endpoint, and b) the target outcome(s) whose intervention effect is being substituted for.	
	**Introduction**		
Background and objectives	2a	Scientific background and explanation of the rationale	
	2b	Specific objectives or hypotheses	
	2.1	State a) that the primary outcome is a surrogate endpoint, and b) the target outcome(s) whose intervention effect is being substituted for (when reporting 2a or 2b)	
	**Methods**		
Trial design	3a	Description of trial design (such as parallel, factorial), including allocation ratio	
	3b	Important changes to methods after trial commencement (such as eligibility criteria), with reasons	
Participants	4a	Eligibility criteria for participants	
	4b	Settings and locations where the data were collected	
Interventions	5	The interventions for each group with sufficient details to allow replication, including how and when they were actually administered	
Outcomes	6a	Completely defined pre-specified primary and secondary outcome measures, including how and when they were assessed	
	6a.1	State the practical or scientific reason(s) for using a surrogate endpoint as a primary outcome	
	6a.2	Justification for selected surrogate: a) evidence (or lack thereof) of surrogate endpoint validation; and b) evidence (or lack thereof) of validity being specific to setting and context used e.g., intervention; disease; population.	
	6b	Any changes to trial outcomes after the trial commenced, with reasons	
Sample size	7a	How sample size was determined	
	7a.1	Clarify if sample size was estimated to demonstrate that a minimum effect on the surrogate endpoint would be predictive of a benefit on the target outcome(s).	
	7b	When applicable, explanation of any interim analyses and stopping guidelines	
Randomisation:			

Sequence generation	8a	Method used to generate the random allocation sequence	
	8b	Type of randomisation; details of any restriction (such as blocking and block size)	
Allocation concealment mechanism	9	Mechanism used to implement the random allocation sequence (such as sequentially numbered containers), describing any steps taken to conceal the sequence until interventions were assigned	
Implementation	10	Who generated the random allocation sequence, who enrolled participants, and who assigned participants to interventions	
Blinding	11a	If done, who was blinded after assignment to interventions (for example, participants, care providers, those assessing outcomes), and how	
	11b	If relevant, a description of the similarity of interventions	
Statistical methods	12a	Statistical methods used to compare groups for primary and secondary outcomes	
	12b	Methods for additional analyses, such as subgroup analyses and adjusted analyses	
	**Results**		
Participant flow (a diagram is strongly recommended)	13a	For each group, the numbers of participants who were randomly assigned received intended treatment and were analysed for the primary outcome	
	13b	For each group, losses, and exclusions after randomisation, together with reasons	
Recruitment	14a	Dates defining the periods of recruitment and follow-up	
	14b	Why the trial ended or was stopped	
Baseline data	15	A table showing baseline demographic and clinical characteristics for each group	
Numbers analysed	16	For each group, the number of participants (denominator) included in each analysis and whether the analysis was by original assigned groups	
Outcomes and estimation	17a	For each primary and secondary outcome, results for each group, and the estimated effect size and its precision (such as 95% confidence interval)	
	17a.1	If the primary outcome is a composite outcome that includes a surrogate endpoint; report the intervention effect on all components.	
	17b	For binary outcomes, the presentation of both absolute and relative effect sizes is recommended	
Ancillary analyses	18	Results of any other analyses performed, including subgroup analyses and adjusted analyses, distinguishing pre-specified from exploratory	
Harms	19	All important harms or unintended effects in each group (for specific guidance, see CONSORT for harms)	.
	**Discussion**		
Limitations	20	Trial limitations, addressing sources of potential bias, imprecision, and, if relevant, the multiplicity of analyses	
Generalisability	21	Generalisability (external validity, applicability) of the trial findings	
Interpretation	22	Interpretation consistent with results, balancing benefits and harms, and considering other relevant evidence	

	22.1	Interpretation of findings of the trial in the context of using a surrogate primary endpoint, including its known validity for intervention effects on the target outcome and the potential benefit-risk assessments of the tested intervention for participants.	
	22.2	Comment on whether the trial design (including sample size and follow-up period), given the use of a surrogate endpoint, adequately captures the potential harms of the intervention being tested.	
	22.3	State what the plans are to conduct subsequent analyses/studies to verify current findings on the target outcome(s).	
**Other information**			
Registration	23	Registration number and name of trial registry	
Protocol	24	Where the full trial protocol can be accessed, if available	
Funding	25	Sources of funding and other support (such as a supply of drugs), the role of funders	
**New items**			
Participant engagement	26.1	State whether and how trial participants were engaged and informed before enrolment that the trial was designed to evaluate an intervention's effect using a surrogate endpoint.	
Data access	26.2	If surrogate endpoint and target outcome data were collected in the trial, state the open access arrangements for the data for future secondary research.	

Please cite as: Manyara AM, Davies P, Stewart D, et al. Reporting of surrogate endpoints in randomised controlled trial reports (CONSORT-Surrogate): extension checklist with explanation and elaboration. *BMJ* 2024;386:e078524. doi:10.1136/bmj-2023-078524.
